# Functional differences between lower limbs during gait in relation to fall risk

**DOI:** 10.3389/fphys.2026.1743503

**Published:** 2026-07-03

**Authors:** Zdenek Svoboda, Lucia Bizovska, Miroslav Janura

**Affiliations:** Department of Natural Sciences in Kinanthropology, Faculty of Physical Culture, Palacky University Olomouc, Olomouc, Czechia

**Keywords:** footedness, laterality, local dynamic stability, Lyapunov exponent, walking

## Abstract

**Introduction:**

The scientific literature suggests a functional asymmetry between the lower limbs during gait, with the non-dominant limb serving a stabilizing role and the dominant limb a leading role. However, it remains unclear whether this asymmetry is also reflected in local dynamic stability, which has been shown to be significantly related to fall risk. This study aimed to assess differences in local dynamic stability between the dominant and non-dominant limbs in relation to fall occurrence.

**Methods:**

The study sample consisted of 131 elderly participants (108 females and 23 males; mean age = 71.0 ± 6.8 years). Local dynamic stability was evaluated using accelerometers attached to the shanks. Outcome variables included short-term and long-term Lyapunov exponents (LE) calculated independently for the vertical, medial–lateral, and anterior–posterior directions. Fall occurrence was prospectively recorded over a one-year period through biweekly phone interviews. After one year, participants were divided into two groups: fallers (at least one fall) and non-fallers (no fall).

**Results:**

Significant asymmetries between the dominant and non-dominant limbs were observed in several local dynamic stability parameters. In particular, the non-dominant limb demonstrated better stability in the medial–lateral direction, whereas the dominant limb showed lower LE values in the anterior–posterior direction. No significant main effect of group was observed. However, a significant limb × group interaction was found for long-term LE in the medial–lateral direction, suggesting altered asymmetry patterns in fallers compared to non-fallers.

**Discussion:**

In conclusion, this study confirmed the existence of functional asymmetry between limbs during gait in measures of local dynamic stability. The findings suggest that the roles of the limbs are not associated with acute responses but rather with long-term adaptations to perturbations.

## 1 Introduction

Functional differences between the lower limbs in various motor tasks, also referred to as footedness, have been observed in numerous studies. The limb (foot) that is preferentially used to manipulate an object or to lead out, as in jumping, is considered the preferred limb, whereas the limb that supports the activities of the preferred foot by providing postural and stabilizing support is regarded as the non-preferred limb (Peters, 1988). If a difference in limb performance is observed, irrespective of any pre-existing preference, the limb demonstrating superior performance can be classified as dominant, while the limb exhibiting inferior performance can be considered non-dominant (Peters, 1988; Zverev, 2006).

During certain motor tasks, such as normal walking, the lower limbs exhibit similar movement patterns with only a temporal phase shift, resulting in an almost symmetrical pattern of movement. However, many authors have reported asymmetrical behavior of the lower limbs during able-bodied walking, and thus it is necessary to consider a certain range of functional differences between the lower limbs as a natural characteristic of human gait (Gao et al., 2023; Lathrop-Lambach et al., 2014; Maupas et al., 1999; Riskowski et al., 2012; Sadeghi et al., 2000).

One hypothesis explaining gait asymmetry proposes that the dominant limb primarily contributes to forward progression, whereas the non-dominant limb is more involved in postural control and stabilization (Zverev, 2006). Several studies supported this functional differentiation. Previous research reported greater propulsive loading, higher plantar flexion torque, and increased hip power generation in the dominant limb, while the non-dominant limb demonstrated characteristics associated with stabilization, including greater braking-related loading and lower center of pressure variability during weight acceptance (Balkó et al., 2022; Sadeghi et al., 2000; Svoboda et al., 2015; Valderrabano et al., 2007; Wang and Watanabe, 2012).

Recent studies have highlighted several objective approaches for distinguishing fallers from non-fallers. Ejupi et al. (2017) demonstrated that wearable sensor-based sit-to-stand assessment can reliably detect movement differences between older fallers and non-fallers. Similarly, Zhu et al. (2025) reported that reduced lower-limb power, particularly during leg-press and sit-to-stand tasks, is associated with prospective falls in community-dwelling older adults. In gait analysis, Ehrhardt et al. (2020) showed that a combination of spatio-temporal gait parameters, including variability and asymmetry measures, can distinguish fallers from non-fallers in neurological patients, highlighting the importance of objective instrumented gait symmetry assessment. In addition, Maqbool et al. (2024) demonstrated that gait asymmetry may vary across different phases of the gait cycle.

Although these studies suggest functional differences between the limbs as leading and supporting, the relationship between their roles and the maintenance of dynamic balance during gait is not well documented. This issue may be relevant in relation to fall risk, as understanding the functional roles of the limbs during gait could contribute to a better understanding of balance control mechanisms in older adults. Some authors have suggested that asymmetry analyses during gait may provide additional insights into the specific deficits in balance control mechanisms that contribute to increased gait variability (Peterka et al., 2023).

For assessing dynamic stability, local dynamic stability based on short-term and long-term Lyapunov exponents has commonly been used. Local dynamic stability refers to the sensitivity of the system to infinitesimal perturbations (Dingwell et al., 2001). Several studies have demonstrated a significant relationship between local dynamic stability and falls (Bizovska et al., 2018; Toebes et al., 2012). However, these studies evaluated the local dynamic stability of the center of mass or its approximation using an accelerometer placed on the trunk. Direct evaluation of local dynamic stability in the lower limbs could provide new insights into movement behavior during gait.

To the best of our knowledge, no previous study has investigated differences in local dynamic stability between the dominant and non-dominant limbs during gait. Furthermore, examining the relationship between the functional roles of the limbs during gait and the occurrence of falls may provide a more comprehensive understanding of dynamic gait stability, with potential implications for clinical practice. Thus, the aim of this study is to assess differences in local dynamic stability between the dominant and non-dominant limbs in relation to fall occurrence. We hypothesized that the non-dominant limb would exhibit better local dynamic stability compared to the dominant limb and that this asymmetry might differ between non-fallers and fallers.

## 2 Materials and methods

### 2.1 Sample

This study employed a prospective approach. The study protocol was approved by the Ethics Committee of the Faculty of Physical Culture, Palacky University Olomouc, Olomouc, Czech Republic, no. 24/2014), and all participants provided written informed consent.

Subjects were recruited from local senior clubs through personal communication and flyers. Inclusion criteria were age 60 years or older and the ability to walk without aid for at least 10 min, including turns. Exclusion criteria included neurological or musculoskeletal disorders, and any injury or surgery of the lower limbs within the two years preceding the measurement. The final sample consisted of 131 elderly participants (108 women, 23 men) with a mean age of 71.0 ± 6.8 years, a mean height of 162.5 ± 7.5 cm, and a mean body weight of 75.4 ± 13.6 kg.

### 2.2 Procedures

During baseline measurements, participants completed a questionnaire to verify their eligibility based on the inclusion and exclusion criteria. Limb dominance was determined using three simple mobilizing tasks: the first step upstairs, the first step during walking, and kicking a ball. Based on this assessment, 111 participants were classified as right-limb dominant and 20 participants as left-limb dominant. Participants with ambiguous dominance classification across the assessment tasks were excluded from the study. Local dynamic stability was assessed during 5 min of indoor walking (over ground) in a 25-m-long, well-lit corridor. Participants were instructed to walk at their self-selected speed, proceed straight forward, turn upon reaching a marker on the floor, and continue walking at the same speed to the next turn. Participants performed the walking trials wearing their usual comfortable sports footwear. If corrective visual aids (glasses or contact lenses) were normally used, participants wore them during testing. Measurements were performed during standard daytime hours (morning or afternoon). Before testing, participants were given sufficient time for rest to minimize potential fatigue effects, and the relatively short duration of the walking protocol (5 minutes) was not expected to induce substantial fatigue.

Before walking, 3D accelerometers (sampling rate 296.3 Hz; Trigno wireless system, Delsys Inc., Natick, MA, USA) were attached to both shanks approximately 15 cm above the lateral malleolus. Acceleration was recorded in the vertical (V), medial–lateral (ML), and anterior–posterior (AP) directions. Local dynamic stability was evaluated using short-term and long-term Lyapunov exponents, computed from 150 walking strides for each direction independently.

The Lyapunov exponents quantify the sensitivity of the locomotor system to small perturbations by measuring the rate of divergence of neighboring trajectories in the reconstructed state space. Higher Lyapunov exponent values indicate lower local dynamic stability and a reduced ability to attenuate small perturbations during gait. Short-term Lyapunov exponents are generally interpreted as reflecting the response of the locomotor system to perturbations over a short time scale (approximately one step), whereas long-term Lyapunov exponents describe divergence behavior over multiple subsequent strides.

Prior to analysis, the first 300 samples of the recorded signal were excluded because of sensor stabilization, and strides associated with turning (the last stride before the turn, the turn itself, and the first stride after the turn) were removed to retain only straight walking episodes. The unfiltered acceleration signal was subsequently analyzed. Heel strikes were identified from the anterior–posterior acceleration signal, and 150 consecutive strides were used for the computation of local dynamic stability to ensure reliable estimation of the indices. The selection of 150 strides was based on previous studies reporting that local dynamic stability measures, particularly long-term Lyapunov exponents, require a relatively large number of strides for reliable estimation (Riva et al., 2014). At the same time, the duration of the walking trial (5 minutes) was considered feasible and well tolerated for the older adult population included in the present study.

The resulting time series was resampled to approximately 100 samples per stride. State-space reconstruction was performed using the time-delay embedding method. Time delays were determined from the first minimum of the average mutual information function and were set to 9, 6, and 11 samples for the shank acceleration signals in the vertical, medial–lateral, and anterior–posterior directions, respectively. The embedding dimension was set to 6 based on global false nearest neighbor analysis. Short-term and long-term Lyapunov exponents were then computed using the algorithm proposed by Rosenstein et al., following the methodological procedure described by Riva et al. (2014) and Bizovska et al. (2018).

Fall occurrence was monitored for one year after baseline measurement, in accordance with the recommendations of The Prevention of Falls Network Europe (Lamb et al., 2005). A fall was defined as ‘an unexpected event in which the participants come to rest on the ground, floor, or lower level’. All participants were contacted every two weeks by phone to report any fall, including slips and trips in which they lost their balance and landed on the floor, ground, or a lower level. If a fall was mentioned, additional information was collected regarding the circumstances of the fall, such as location and activity. The collected information regarding fall circumstances was used primarily to verify whether the reported event fulfilled the study definition of a fall and met the predefined inclusion criteria for fall registration. Falls related to sports activities, caused by major external forces, or occurring under impaired visual conditions, were excluded. After one year, participants were divided into two groups: non-fallers (those who had no falls) and fallers (those who had at least one fall).

### 2.3 Data processing

Local dynamic stability was evaluated using short-term and long-term Lyapunov exponents, computed from 150 strides for each direction independently (Riva et al., 2014). The procedures described by Bizovska et al. (2018) were followed.

All statistical analyses were performed using Statistica software (version 12, StatSoft, Inc., Tulsa, OK, USA). Normality of data distributions was assessed using the Kolmogorov–Smirnov test. Basic characteristics of the groups were compared using unpaired t-tests. The effects of limb (dominant, non-dominant), group (fallers, non-fallers), and their interaction (limb × group) were assessed using a two-way mixed design analysis of variance (ANOVA) followed by Tukey *post hoc* tests. To assess the potential influence of confounding factors on local dynamic stability measures, an additional sensitivity analysis was performed. The effects of limb, group, and their interaction were re-evaluated after adjustment for age, sex, BMI, and walking speed. Sex was included as a categorical factor, whereas age, BMI, and walking speed were included as continuous covariates. The sensitivity analysis was performed using a general linear model for repeated measures with the same within-subject (limb) and between-subject (group) structure as in the primary analysis.

The significance level was set at α = 0.05. Owing to the evaluation of multiple directions, a Bonferroni correction was applied, resulting in an adjusted significance level of 0.05/3 = 0.017. Effect size was assessed using partial η^2^, with values of 0.01, 0.06, and 0.14 interpreted as small, medium, and large effects, respectively (Cohen, 1988).

## 3 Results

As described above, after one year of fall observation, the sample was divided into fallers (at least one fall; 5 males, 45 females) and non-fallers (no falls; 18 males, 63 females). A basic description of the groups, along with the results of statistical comparisons between them (p-values), is presented in **Table 1**. During the one-year follow-up period, a total of 75 falls were recorded among the 50 fallers. Of these participants, 34 experienced a single fall, whereas 16 participants reported recurrent falls (≥2 falls). The number of falls per participant ranged from 1 to 4 falls.

**Table 1:** Basic characteristics of the groups.

Variable	Non-fallers (n = 81)		Fallers (n = 50)		P value
	Mean	SD	Mean	SD	
Age [years]	70.5	6.4	71.7	7.3	0.364
Body height [cm]	163.6	7.8	160.8	6.7	0.037
Body weight [kg]	77.5	14.8	72.0	10.8	0.025
BMI [kg.m-2]	28.8	4.6	27.9	4.6	0.273
Walking speed [m.s-1]	1.25	0.16	1.23	0.15	0.656

SD, standard deviation; BMI, body mass index.

Evaluation of local dynamic stability showed no significant effect of group (fallers, non-fallers). The effect of limb (dominant, non-dominant) was significant for the long-term Lyapunov exponent in ML (lower values in the non-dominant limb, medium effect) and AP (lower values in the dominant limb, large effect) directions (**Table 2**). *Post hoc* comparisons indicated significant differences between limbs for the ML direction only in fallers (p < 0.001), whereas for the AP direction, significant differences were observed in both fallers (p = 0.015) and non-fallers (p < 0.001). The effect of limb was also significant for the short-term Lyapunov exponent in the AP direction (lower values in the dominant limb, small effect), but *post hoc* comparisons revealed no significant differences between limbs within individual groups.

**Table 2:** Lyapunov exponents for dominant and non-dominant limbs in groups of fallers and non-fallers.

Variable	Limb	Dominant		Non-dominant		P value (η2)		
	Group	Mean	SD	Mean	SD	Effect of group	Effect of limb	Limb × group effect
ltLE_V	Non-fallers	0.025	0.011	0.025	0.011	0.974 (<0.01)	0.870 (<0.01)	0.957 (<0.01)
	Fallers	0.025	0.008	0.025	0.008			
ltLE_ML	Non-fallers	0.016	0.007	0.015	0.007	0.901 (<0.01)	<0.001 (0.121)	0.011 (0.049)
	Fallers	0.017	0.007	0.014	0.005			
ltLE_AP	Non-fallers	0.030	0.011	0.033	0.011	0.720 (<0.01)	<0.001 (0.161)	0.788 (<0.01)
	Fallers	0.031	0.008	0.033	0.008			
stLE_V	Non-fallers	1.138	0.144	1.147	0.153	0.777 (<0.01)	0.972 (<0.01)	0.277 (<0.01)
	Fallers	1.155	0.157	1.145	0.171			
stLE_ML	Non-fallers	1.243	0.223	1.219	0.236	0.448 (<0.01)	0.273 (<0.01)	0.723 (<0.01)
	Fallers	1.264	0.185	1.251	0.197			
stLE_AP	Non-fallers	0.830	0.156	0.851	0.169	0.906 (<0.01)	0.005 (0.059)	0.495 (<0.01)
	Fallers	0.827	0.131	0.860	0.130			

SD, standard deviation; ltLE, long-term Lyapunov exponent; stLE, short-term Lyapunov exponent; V, vertical direction; ML, medial–lateral direction; AP, anterior–posterior direction; for η^2^ only values greater than 0.01 (small, medium or large effect) are presented.

The limb × group interaction effect was significant only for the long-term Lyapunov exponent in the medial-lateral direction (small effect). Pairwise comparisons showed a significant difference between limbs in the fallers group, whereas no significant difference was observed in the non-fallers group.

An additional sensitivity analysis adjusted for age, sex, BMI, and walking speed showed that several local dynamic stability parameters were influenced by these covariates. However, the significant limb × group interaction for long-term Lyapunov exponent in the medial–lateral direction remained present after adjustment.

## 4 Discussion

The main purpose of our study was to investigate whether functional asymmetry exists between limbs during gait with regard to local dynamic stability and fall risk. Our results demonstrated that local dynamic stability differs between limbs. In the non-dominant limb, smaller values in the medial–lateral direction suggest better stability, while in the dominant limb, lower values in the anterior–posterior direction may reflect smoother propulsion -related control.

In general, many studies have reported that asymmetry reflects a natural functional difference between the limbs, and that this difference is not a consequence of abnormality but rather relates to the specific contributions of each limb to propulsion and control tasks (Echeverria et al., 2010). It is important to note, however, that limb dominance is not constant and can exhibit a range of dynamic behaviors, from synchronization to switching of the dominant limb (Echeverria et al., 2010).

During walking, some studies suggest that the non-dominant limb primarily secures or stabilizes the body (Nagano et al., 2011), whereas the dominant limb contributes more to forward progression. From this perspective, it is important to recognize between variables that describe movement or interaction between the body and the ground in the medial-lateral and anterior-posterior directions. Many studies have demonstrated that maintaining gait stability in the medial-lateral direction is important for balance control and may be associated with fall risk. Therefore, the lower medial-lateral Lyapunov exponent values observed in the non-dominant limb may reflect a more stabilizing functional role of this limb during gait.

In our study, this corresponds to the non-dominant limb, which demonstrated lower long-term Lyapunov exponent values, indicating better stability. Conversely, smaller values in the anterior–posterior direction were observed in the dominant limb compared to the non-dominant limb in both long-term and short-term Lyapunov exponents. These findings are consistent with those reported in the literature. Previous studies have shown that the non-dominant limb is characterized by greater load (Balkó et al., 2022) and lower center of pressure movement variability (Svoboda et al., 2015) during gait phases associated with limb stabilization. In contrast, the dominant limb exhibits greater load (Balkó et al., 2022), higher center of pressure velocity in the anterior–posterior direction (Wang and Watanabe, 2012), and lower center of pressure variability during propulsion. Further evidence supporting the leading role of the dominant limb includes greater plantarflexion torque (Valderrabano et al., 2007) and increased power generation at the hip during flexion (Sadeghi et al., 1997).

It can be expected that various factors may influence functional asymmetry between limbs during gait and other mobility tasks. In general, more demanding tasks appear to result in greater differences between limbs. Seeley et al. (2008) found significant differences in force impulses between limbs only at fast gait speeds, with no differences observed at slow or preferred speeds. Similarly, Guo et al. (2024) reported that differences in parameters related to the center of plantar pressure movement of the leading foot during descent of a single step increased with higher step heights. In our study, participants walked at their self-selected speed, which corresponds to the slow speed described by (Seeley et al., 2008); however, their subjects were young adults. In our group of older adults, this self-selected walking speed appears to be sufficiently demanding to elicit distinct functional roles of the limbs.

In relation to fall risk, local dynamic stability parameters, especially the short-term Lyapunov exponent, have shown potential for distinguishing between fallers and non-fallers (Bizovska et al., 2018; Toebes et al., 2012). In our study, no significant differences were observed in these variables, suggesting that the different functional roles of the limbs are not strongly associated with actual responses to perturbations. The lower short-term Lyapunov exponent values observed in the anterior–posterior direction in the dominant limb may be related to more stable forward progression rather than to greater stabilizing ability of the limb. However, these interpretations should be considered cautiously, as the present study did not directly assess muscle activity, kinetics, or propulsion-related biomechanical variables.

Furthermore, we did not observe any significant differences between fallers and non-fallers, even in the short-term Lyapunov exponents. This discrepancy may be explained by the placement of the accelerometers. In the aforementioned studies, accelerometers were placed on the trunk, whereas in our study, they were attached to the shanks. The sensitivity analysis further indicated that local dynamic stability measures may be substantially influenced by factors such as walking speed, BMI, and sex, which is consistent with previous findings emphasizing the multifactorial nature of gait stability in older adults. On the other hand, our results suggest that the dominant and non-dominant limbs may differ primarily in divergence behavior observed over subsequent strides rather than over a single step, as evidenced by significant differences in long-term Lyapunov exponents, which are calculated between the 4th and 10th stride. In the medial–lateral direction, values were smaller for the non-dominant limb, whereas in the anterior–posterior direction, for the dominant limb, suggesting findings consistent with the hypothesis of leading and stabilizing limb functions during gait. On the other hand, it should be considered that humans must adapt these parameters at every step to respond to exogenous and/or endogenic perturbations, employing both inter-leg and intra-leg control (Kozlowska et al., 2017). Consequently, in some cases, functional asymmetry may be inhibited owing to the limited time available for response. However, the interpretation of short-term and long-term Lyapunov exponents in relation to specific physiological control mechanisms remains partly theoretical and should therefore be interpreted cautiously.

Importantly, our findings provide stronger evidence for the existence of functional asymmetry between limbs during gait than for a direct association between this asymmetry and fall risk. Although a significant interaction effect was observed for long-term local dynamic stability in the medial–lateral direction, the absence of significant group effects in most parameters suggests that the relationship between limb asymmetry and fall occurrence is limited.

Our study has several limitations. Although the recruitment procedure targeted both males and females, the number of male participants was considerably lower. This imbalance may be explained by the greater interest of older females in participating in scientific studies related to balance evaluation. Similarly, the ratio of males to females differed between the faller and non-faller groups. Unfortunately, this could not be influenced, as it reflects the actual occurrence of falls. Another limitation is that gait assessment was performed under controlled laboratory conditions, which may not fully reflect gait behavior during daily-life activities. Finally, although falls were prospectively monitored using regular telephone follow-up, minor fall events may still have been underreported. Although information regarding fall circumstances was collected to verify fall events, detailed analyses of fall mechanisms, severity, and contextual characteristics were beyond the scope of the present study.

We acknowledge that several additional factors potentially associated with gait stability and fall risk were not systematically assessed in the present study. Physical activity level and cognitive function were not evaluated. Information regarding medication use was collected primarily to exclude participants using medication substantially affecting balance or gait; however, medication use was not included in the statistical analysis. Previous fall history was also recorded, but because of its low occurrence and highly skewed distribution in the present sample, this variable was not included as a covariate in the sensitivity analysis.

Previous studies have demonstrated moderate to good reliability of gait parameters derived from wearable inertial sensors (Kobsar et al., 2020), as well as acceptable reliability of local dynamic stability measures during walking (van Schooten et al., 2013). However, most available reliability studies have focused primarily on trunk-mounted sensors or on spatio-temporal gait parameters rather than local dynamic stability assessed from shank-mounted sensors. Therefore, although the present methodology was based on standardized inertial sensor procedures, the specific test–retest reliability and measurement error characteristics of shank-based local dynamic stability assessment should be further investigated in future studies.

In conclusion, our study demonstrated functional asymmetry in local dynamic stability between the limbs during walking in older adults. The observed differences are consistent with the hypothesis that the non-dominant limb may contribute more to stabilization, whereas the dominant limb may be more involved in forward progression. However, the association between these asymmetries and fall risk appears limited, as significant differences between fallers and non-fallers were observed only for one local dynamic stability parameter.

## Data Availability

The datasets generated for this study can be found in the Zenodo https://doi.org/10.5281/zenodo.20430175.
